# Public health round-up

**DOI:** 10.2471/BLT.21.010621

**Published:** 2021-06-01

**International Nurses Day**

A nurse treats a 3-year-old girl for a severe ear infection at the Lukomba Rural Health Centre in Kapiri Mposhi District, Zambia. Last month the World Health Organization marked International Nurses Day on 12 May with the launch of a new report entitled *The state of the world’s nursing 2020. *

**Figure:**
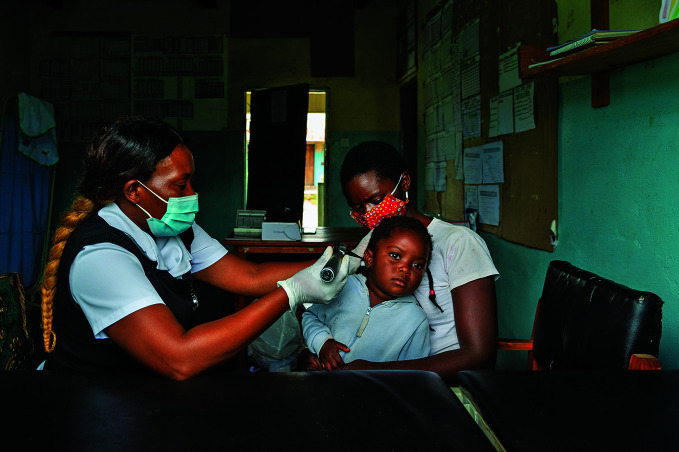
WHO/Blink Media/Gareth Bentley

## Another COVID-19 vaccine listed for emergency use

The World Health Organization (WHO) listed the Sinopharm vaccine for coronavirus disease 2019 (COVID-19) for emergency use, giving the green light for this vaccine to be rolled out globally. 

The Sinopharm vaccine is produced by Beijing Bio-Institute of Biological Products Co Ltd, a subsidiary of China National Biotec Group, and joins four other COVID-19 vaccines (Pfizer/BioNTech, AstraZeneca/Oxford, Janssen and Moderna) to be approved for emergency use by WHO. 

“The addition of this vaccine has the potential to rapidly accelerate COVID-19 vaccine access for countries seeking to protect health workers and populations at risk,” said Dr Mariângela Simão, WHO Assistant Director-General for Access to Health Products. 

https://bit.ly/3tL1dNz

## Global report on nurses

More than five million more nurses are needed in 191 countries, with the biggest gaps in Africa, South-East Asia, the WHO Eastern Mediterranean Region and parts of Latin America, according to a new WHO report. 

The report found that while nursing numbers had increased by 4.7 million between 2013 and 2018, there was still a shortfall of 5.9 million in 2018. Currently, there are just under 28 million nurses working worldwide.

*State of the world's nursing 2020: investing in education, jobs and leadership *was prepared**by WHO in partnership with the International Council of Nurses. 

Nurses account for more than half of all the world’s health workers, providing vital services throughout the health system. Around the world they have played a prominent role in countries’ response to the COVID-19 pandemic.

https://bit.ly/3fjhRPj

## Intellectual property and COVID-19 vaccines

The United States government pledged to support a temporary waiver of intellectual property on COVID-19 vaccines to expedite their production and distribution in low- and middle-income countries in a statement on 5 May.

WHO Director-General Tedros Adhanom Ghebreyesus praised the commitment as “a monumental moment in the fight against COVID-19”, calling it “a powerful example of American leadership to address global health challenges”.

The US government said that the extraordinary circumstances caused by the COVID-19 pandemic required extraordinary measures and intellectual property protections on vaccines should be waived to help end the pandemic. 

https://bit.ly/2SNYJ4g

## WHO Hub for Pandemic and Epidemic Intelligence

A new global hub for pandemic and epidemic intelligence, data, surveillance and analytics innovation will be established in Berlin, WHO and the German government announced on 5 May.

Working with partners from across the world, the WHO Hub for Pandemic and Epidemic Intelligence will lead innovations in data analytics across the largest network of global data to predict, prevent, detect, prepare for and respond to pandemic and epidemic risks.

“The current COVID-19 pandemic has taught us that we can only fight pandemics and epidemics together,” said German Chancellor Dr Angela Merkel. “The new WHO Hub will be a global platform for pandemic prevention, bringing together various governmental, academic and private sector institutions.”

https://bit.ly/3eIvx7m

## Moderna vaccine approved for emergency use

WHO listed the Moderna COVID-19 vaccine (mRNA 1273) for emergency use on 30 April. 

WHO’s Emergency Use Listing assesses the quality, safety and efficacy of COVID-19 vaccines and is a prerequisite for inclusion in the COVAX Facility vaccine supply. Such listing also allows countries to expedite their own regulatory approval to import and administer COVID-19 vaccines.

The Moderna vaccine is an mRNA-based vaccine. It was found by WHO’s Strategic Advisory Group of Experts on Immunization to have an efficacy of 94.1%, based on a median follow-up of two months. Although the vaccine is provided as a frozen suspension at −25°C to –15°C in a multidose vial, vials can be stored at 2–8°C for up to 30 days prior to withdrawal of the first dose, meaning that ultra-cold chain equipment may not always be necessary to deploy the vaccine. 

https://bit.ly/3oaxP2b

## Disruptions to health services

Substantial disruptions to essential health services persist as health systems struggle to respond to the COVID-19 pandemic, according to the results of the second round of a WHO pulse survey released on 23 April.

The survey shows that 94% of countries (127 of 135) that participated in the survey reported one or more disruptions to essential health services, marking no substantial global change since the first such survey conducted in the summer of 2020. Within countries, however, the magnitude and extent of disruptions has generally decreased. 

https://bit.ly/2RPeyHk

## Sweden donates vaccines 

The Swedish government announced that it would donate 1 million doses of the AstraZeneca vaccine to the COVAX Facility to provide life-saving vaccines to people at risk from COVID-19 in low-income countries.

The announcement on 3 May was welcomed by WHO Director-General Tedros Adhanom Ghebreyesus who described it as “a superb gesture” that should be followed by governments around the world to accelerate the equitable rollout of vaccines globally.

https://bit.ly/3boUmD1

## ACT-Accelerator 12 months on 

WHO warned world leaders that unless they invest more in saving lives by treating the cause of the COVID-19 pandemic now, their countries may continue to spend vast sums of money on the human, social and economic consequences of the pandemic. 

The Access to COVID-19 Tools (ACT) Accelerator collaboration started working in April 2020 to develop and deliver the tests, treatments and vaccines that the world needs to fight COVID-19. 

A report released in April, entitled *ACT now, ACT together 2020-2021 impact report*, found that the collaboration had made progress in expediting research and development and in terms of fund raising. 

Governments and private sector funders contributed US$ 14.1 billion in ACT-Accelerator’s first 12 months of operation and galvanized research but the new tests, treatments and vaccines for COVID-19 had mainly been distributed in high-income countries. 

And the collaboration still faced a funding gap of US$ 19 billion. 

“We call on all nations to come together in global solidarity. It isn’t just the right thing to do, it is also the fastest and most effective way to save lives, protect health systems and restore economies,” said WHO Director-General Tedros Adhanom Ghebreyesus.

“We cannot defeat this virus one country or region at a time. We can only do it with a coordinated global effort, based on the principles of solidarity, equity and sharing.”

https://bit.ly/3bnl4ff

## Immunization services 

Immunization services have started to recover from disruptions caused by COVID-19 epidemics, but millions of children still remain vulnerable to deadly vaccine-preventable diseases, WHO, the United Nations Children’s Fund and Gavi, the Vaccine Alliance warned.

To help tackle these challenges, WHO and its partners launched the Immunization Agenda 2030 (IA2030), a new global strategy to create stronger immunization systems.

The Agenda focuses on vaccination throughout life, from infancy through to adolescence and older age. 

The targets to be reached by 2030 include achieving 90% coverage for essential vaccines given in childhood and adolescence, halving the number of children completely missing out on vaccines and completing 500 national or subnational introductions of new or under-utilized vaccines, such as those for COVID-19, rotavirus or human papillomavirus.

https://bit.ly/2RRAL7v

**Cover photo**

Health workers visit a nomad family in their yurt in Darhan-Uul Province, Mongolia.

**Figure:**
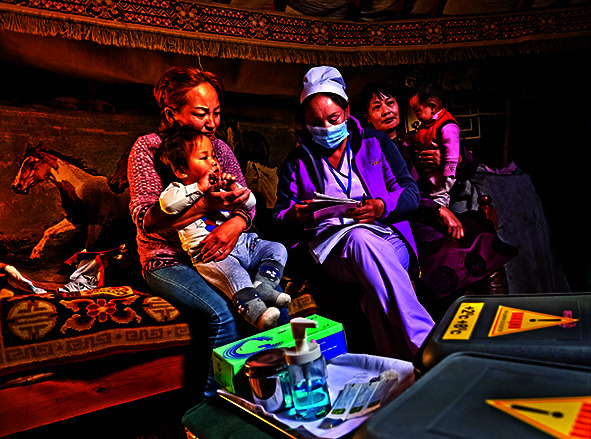
WHO/Yoshi Shimizu

## New regulatory guidance

WHO launched new guidance on regulatory system strengthening activities, including regulatory cooperation, convergence and transparency.

Launched 29 April, the guidance was published as Annexes 10 and 11 in the 55th report of the WHO Expert Committee on Specifications for Pharmaceutical Preparations. 

The aim of the guidance is to support countries in improving the oversight and regulation of medicines and health products, and to promote greater collaboration between regulators regionally and internationally to leverage resources more efficiently and ensure quality health products reach people faster.

https://bit.ly/2RQdL8Y

**Looking ahead**

11–13 June, Carbis Bay, the United Kingdom: 47th Group of Seven countries (G7) summit.

14­–30 September, New York: United Nations General Assembly.

